# Experimental and Computational Investigation of the IDDSI Flow Test of Liquids Used in Dysphagia Management

**DOI:** 10.1007/s10439-019-02308-y

**Published:** 2019-06-19

**Authors:** Ben Hanson, Rashid Jamshidi, Andrew Redfearn, Ryan Begley, Catriona M. Steele

**Affiliations:** 1 UCL Mechanical Engineering, Torrington Place, London, WC1E 7JE UK; 2 UCL Chemical Engineering, Torrington Place, London, WC1E 7JE UK; 3 Toronto Rehabilitation Institute – University Health Network, 550 University Avenue, #12-101, Toronto, ON M5G 2A2 Canada; 4 Rehabilitation Sciences Institute, Faculty of Medicine, University of Toronto, 500 University Avenue, Suite 160, Toronto, ON Canada

**Keywords:** Rheology, Fluid dynamics, Swallowing, International Dysphagia Diet Standardisation Initiative, Non-Newtonian, Thickened drinks, Texture modification

## Abstract

The International Dysphagia Diet Standardisation Initiative (IDDSI) flow test, using a standard 10-mL syringe, is being adopted in many countries for clinical measurement of the consistency of drinks. The working hypothesis is that thickening drinks to retard flow can be advantageous for individuals who struggle to cope with thin drinks. This study assesses how the IDDSI test relates to rheology and clinical knowledge of physiological flows during swallowing. With no pre-existing analytical solution for internal flow through the syringe, a computational model was designed, incorporating rheometry data from a variety of Newtonian and non-Newtonian liquids. The computational model was validated experimentally across the range of liquids but the technique showed limitations in simulating dripping and cohesiveness. Gum-based liquids which were strongly shear-thinning (0.12 < *n* < 0.25) showed plug-flow characteristics with 90% of the shear occurring in only 22% of the radial dimension. Shear rates were maximal at the nozzle outlet (> 60 times higher than the barrel) and reached 7400/s for the thinnest gum-based liquid. Shear rheology data alone was unable to describe the flow of these drinks. The flow conditions in the test varied according to the type and consistency of liquid, relating to the desired clinical effect.

## Introduction

During swallowing, the safe and efficient transport of a liquid bolus depends on a biomechanical process involving up to 26 paired muscles whose contraction is tailored to the flow properties of a liquid.^7^ Dysphagia, the term for an impairment to the safety and/or efficiency of swallowing, frequently involves inappropriate timing, strength or coordination of muscle contraction leading to airway invasion or the accumulation of post-swallow residue.^7^,^37^ In such situations, thickened liquids are a widely-used intervention, based on the idea that slower flow of these thicker liquids provides the person with extra time in which to achieve airway closure as well as enabling better control of liquid flow by the tongue and other swallowing muscles.^24^,^32^ In order for texture modification to be effective for people with dysphagia, it is important to use standardized, reliable, controlled textures.^6^ This study investigates the new international standard for texture modification of liquids, assessing its relationship with rheology, and its physiological relevance.

The physiological process of swallowing a liquid involves a wide range of fluid deformations and rates; these are not easily measured *in vivo* and are not well understood even in the healthy swallow. Simulations have been able to demonstrate that a bolus in a squeeze-driven flow will experience maximum shear rates at the interfaces with the tongue or palate which could reach magnitudes of 1000–10,000/s, with 0 shear in the bolus center. Those wall shear rates are strongly dependent on bolus consistency, degree of slip, driving pressure and the gap width.^25^ Dysphagia can affect the swallowing process in many ways depending whether the underlying deficit is sensory, motor and/or structural. Each case requires assessment by a trained clinician (typically a Speech and Language Pathologist) to determine whether any texture modification strategy is effective in managing eating and drinking. Current research using *in vitro* mechatronic swallow simulators is beginning to contribute to the general understanding of fluid dynamics during swallowing, aiming to improve the efficacy of texture modification as a dysphagia intervention.^9^,^29^,^31^ These have provided some data indicating flow speeds decreasing with increased thickness and differences in flow between different material types.^29^ A wide variety of liquid types are used in clinical practice from jelly-like gum-thickened juices to nutritional supplements with high protein and fat content. These have a range of non-Newtonian characteristics^1^,^36^,^38^ meaning their flow behavior *in vitro* depends on many different rheological parameters. That difficulty in objectively classifying “thickness” has been a major challenge to the effective clinical use of texture modification strategies.

In published studies of texture modified products, shear viscosity is the most often-reported measure, typically reporting apparent viscosity at shear rates in the range 1–1000/s.^30^ Shear thinning behavior is almost universal, though the slope of apparent viscosity vs shear rate differs between types of liquid. Thus two liquids with equal apparent viscosity at one shear rate can have very different viscosity at higher and lower shear rates.^21^,^26^,^30^,^40^ Clinically this is important since two types of thickened liquid with the same reported viscosity at one measurement rate may exhibit different clinical outcomes, e.g. being incompletely swallowed.^37^ The flows during swallowing will involve a range of shear rates but the deformations *in vivo* are unlikely to be purely shear as in a rheometer. For this reason, pioneering investigations have recently investigated the potential role of extensional flow behavior, measuring extensional viscosity independently of shear viscosity using filament stretching methods.^11^,^19^,^40^ Dynamic mechanical analysis (DMA) has also been applied to measure viscous and elastic moduli^4^—texture modified materials have been shown to have a significant elastic component which may relate to their cohesiveness when swallowed. Finally, yield stress has been measured^14^,^21^ and although its absolute magnitude is small, it can be sufficient to support surface texture to a height of a few mm which enables semi-fluid foods to be consumed with a fork or spoon. DMA and yield stress measurements involve stresses and strains many orders of magnitude smaller than those involved in swallowing,^13^,^33^ yet the outcomes have been shown to relate to perception.^12^,^19^

Clinical effectiveness depends on a combination of rheological properties, but unfortunately the necessary rheometry instruments are not available outside specialist laboratories. Therefore an objective and practical measurement is required for clinical use. These have been applied elsewhere in food and non-food applications such as paints, oils and building materials. For example, a bucket-sized volume of concrete may be subjected to a “slump test” to assess its consistency.^22^ In food science, a Bostwick Consistometer (CSC Scientific Company, Fairfax, VA, USA) is an assessment of the slump of sauces and condiments, using a volume of 75 mL which is released to flow along a channel. The distance travelled by the liquid over 30 s is used to classify consistency.^2^ An adaptation of slumping has been used with reference to dysphagia drinks, called the line-spread test.^18^ A further practical test involves measuring the speed at which liquid flows through a funnel—the Marsh Funnel^20^ is an implementation developed in the drilling industry, while in the dairy industry a smaller-scale Posthumus Funnel^28^ (Dutch language) is used to classify yoghurts, creams and custards.

The new International Dysphagia Diet Standardisation Initiative (IDDSI) classification system selected an objective but practical measurement for liquids which could be used in kitchens and bedsides as well as laboratories.^5^ The IDDSI flow test is somewhat similar to the aforementioned funnel-based tools and uses a standard 10 mL Luer slip tip syringe as the “funnel”. Rather than measuring the time required for a sample to flow through the syringe, the test classifies consistency based on the volume of the residual liquid in the syringe after a period of 10 s flow. The resulting levels are defined as Level 0 Thin (0–1 mL liquid remaining), Level 1 Slightly-Thick (1–4 mL), Level 2 Mildly Thick (4–8 mL) and Level 3, Moderately Thick (8–10 mL).

Given the complex rheology of texture modified liquids it’s an important challenge to identify how the scalar IDDSI test result (0–10) relates to the various rheological parameters reported previously, and that is the first aim of this study. The test aims to evaluate flow relevant to dysphagia, however the flow conditions within the syringe itself have not been published: that is the second aim. Since it is not possible to measure the flow directly within the syringe, a mathematical model is required; unfortunately, despite the prevalence of syringes there is no previously-published model which describes flow profiles within the body and nozzle of the syringe. Therefore the technical investigation begins with building and validating a fluid dynamics model of gravity-driven flow of non-Newtonian liquids through a 10-mL syringe.

## Materials and Methods

### Mathematical Model

A mathematical analysis of the fluid mechanics within the IDDSI flow test was conducted. The model assumes the liquid contained is subject only to hydrostatic pressure which is proportional to the height of the liquid. The fluid dynamics inside the syringe can be described by the Navier–Stokes equations, a set of continuity and momentum equations covering both fluids in this model: liquid and air [a recent explanation is provided by Batchelor,^3^ for example]. In some simple cases these can be solved analytically; to investigate whether a simplification is possible for this flow test, the process involves comparing the ratio of different forces acting on the liquids and investigating the dominance of each term.

In this model, gravity creates hydrostatic pressure which in turn converts to inertia for the motion which is resisted by viscous (friction) forces exerted by the walls on the liquid. The fluid dynamics of this system can be characterized by two dimensionless numbers (for Newtonian cases): the Froude and Reynolds numbers. The Froude number is the ratio between inertia and gravity forces and is defined as:

1 $$ Fr = \sqrt {\frac{{U_{\text{m}}^{2} }}{gD}} $$

in which $U_{\text{m}}$ is the mean liquid velocity, $g$ is the gravitational acceleration and $D$ is the diameter.

The Reynolds number is the ratio between inertia and viscous forces:

2 $$ Re = \frac{{\rho U_{\text{m}} D}}{\mu } $$

where $\rho$ is the density and $\mu$ is the dynamic viscosity of the liquid.

For this system, the barrel diameter is much larger than the nozzle and hence the flow inside the barrel is much slower than in the nozzle. Thus, the Reynolds number in the barrel is much smaller than inside the nozzle. The effect of gravity is dominant in the barrel, however, inertia is dominant in the nozzle. Using nominal values for water gives a first approximation of the range of these dimensionless numbers: the Froude number can change from 0.02 (gravity dominated) inside the barrel to 5 (inertia dominated) inside the nozzle, and the Reynolds number can change from 100 inside the barrel to 1000 in the nozzle (both inertia dominated). For thicker liquids, both Froude and Reynolds numbers will decrease.

This simple analysis indicated it is not generally possible to neglect any categories of the forces and simplify the fluid motion equations for this system to find an analytical solution. This outcome has also been found earlier by Kutter *et al*., modelling a similar geometry.^16^ In such situations, computational fluid dynamics (CFD) can be applied to find solutions by dividing the geometry into many (usually thousands) discrete, simple elements and solving the dynamic equations simultaneously.

### Computational Model

**Figure 1:**
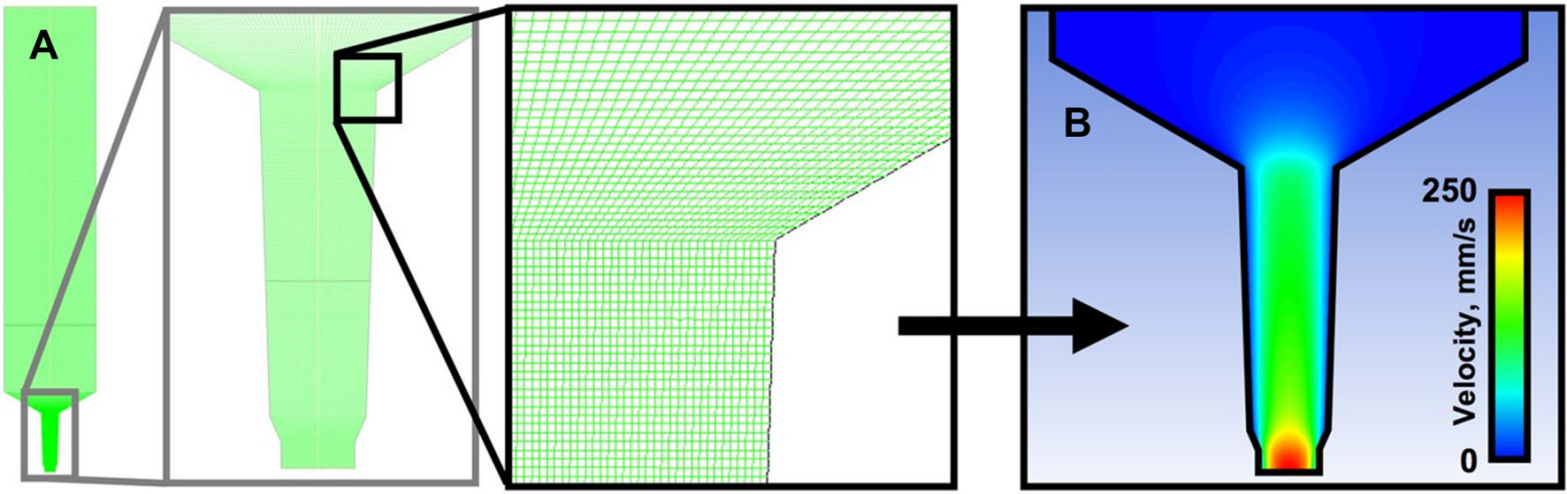
(a) The syringe geometry was discretized into quadrilateral elements. (b) Discrete computation of non-Newtonian fluid models produced maps of fluid velocity, pressure and shear rates; one example velocity map is shown here.

Since the geometry is axially-symmetric throughout and the pressure acts purely along the axial direction it was assumed that the flow inside the syringe was also axially symmetric and hence, the flow motion equations only needed to be solved in a cut-plane of the syringe. This two-dimensional (2D) configuration requires much less computational resources compared to three-dimensions. The 2D geometry of the computational domain is shown in Fig. 1.

The Volume of Fluid (VOF) model was used to track the interface between water and air inside the syringe over time. The tracking of the interfaces between the phases is accomplished by the solution of a continuity equation for the volume fraction of one of the phases. In our case, since the two fluids (liquid and air) are incompressible and there is no mass transfer happening between them, this equation can be simplified as:

3 $$ \frac{\partial \alpha }{\partial t} + \nabla \cdot \alpha \vec{\varvec{U}} = 0 $$

In which $\alpha$ is the volume fraction of the secondary phase (here the liquid) and $\vec{\varvec{U}}$ is its velocity field. The volume fraction of the primary phase (here air) requires no additional equation because the sum of the two volume fractions is under the constraint of $\alpha_{\text{air}} + \alpha_{\text{liquid}} = 1$. Air has the density of $\rho_{\text{air}} = 1.184 {\text{kg}}/{\text{m}}^{3}$ and the dynamic viscosity of $\mu_{\text{air}} = 1.86e - 5 \;{\text{Pa}}\;{\text{s}}$ . For the liquids, the density is calculated by measuring their mass for a specific volume and the viscosity is measured using a shear rheometer (see “Materials” section and “Results” section).

Thickened liquids were created from mixing powdered starch or gum with water until homogeneous (described later in “Thickened Drink Preparation”). The liquids were therefore assumed to comprise uniform continuous media having a no-slip interface with the walls of the geometry. Fluent 17.2 software (CAE Associates, Middlebury, CT, USA) was used to solve the set of continuity, momentum and the volume fraction tracking equations. A grid-independence study was performed using three different numbers of mesh cells. Following this, a structured grid with 7000 quadrilateral cells was selected and all the simulations were carried out using the same grid. At time zero, the computational domain is assumed to be filled with the liquid and as the liquids drains from the nozzle, air replaces it from the top. The top boundary of the domain is assumed a pressure outlet boundary with atmospheric pressure. The circular contact line at the upper liquid / air / solid-wall interface does not move; as the simulated liquid drains, it leaves a film coating on the solid walls. In reality, the residue on the walls is variable in quantity and patterning, however there is not a practical, reliable, computational approach to model a moving interface more realistically.

The exit section of the nozzle is also assumed as a pressure outlet. In the cases where the liquids drain continuously from the nozzle (thinner liquids) it was assumed that as soon as they exit to ambience, the pressure reaches atmospheric. However, in some experiments with thick liquids, the liquids were observed to form a droplet at the exit section of the nozzle and drip instead of continuously flowing. This was observed at Levels 2 and 3 for Starch and Gum-thickened liquids but only at the thickest (Level 3) Glycerol-water mixture. In these cases, the pressure at this boundary is not atmospheric: surface tension and viscous stresses would apply a significant periodic outlet pressure as each droplet is formed and released. Attempting to model individual droplet formation dynamically was rejected on the basis that the additional computational complexity could not be justified by any predicted gain in fidelity: the rheological measurements of these materials could not be assumed to extrapolate to droplet-formation. However, the effect of dripping was included in the model by using a steady-state approximation of the mean boundary pressure. This model has been discussed and validated in similar dripping-mode applications^10^,^41^ where it was judged to give a reasonable accuracy especially at the final stages of droplet formation. In this application, viscous effects on surface tension and droplet curvature change are assumed negligible in comparison to the capillary flow resistance of the nozzle tube. The applicable pressure was calculated by assuming the constant presence of a hypothetical droplet at the exit of the nozzle with a diameter equal to the nozzle. The pressure inside this hypothetical droplet can be calculated using the Young–Laplace equation:

4 $$ P_{\text{droplet}} - P_{\text{atmosphere}} = \frac{2\sigma }{r} $$

where $\sigma$ is the surface tension between the liquids and air and $r$ is the radius of the nozzle at the exit section. The surface tension between the gum-thickened liquids and air decreases slightly as the concentration of the gum increases, but for these concentrations it is very close (within 2%) to the surface tension for water and air at room temperature (0.072 N/m).^11^ Thus, given $r$ = 0.9 mm, the assumed droplet pressure is 160 Pa, which was used as the outlet boundary condition for cases with dripping liquids.

A time interval of 0.01 s was selected as the time step of the transient simulation for the whole 10 sec period of the test, after applying the Courant–Friedrichs–Lewy (CFL) condition and verifying that residual errors were typically < 10^−13^.

The simulation of a fluids’ flow is governed by its rheology, and in this case the dysphagia-management drinks are known to be non-Newtonian. Herschel-Bulkley models have been successfully applied to similar starch- and gum-thickened liquids previously and that model type was adopted here. Model parameters were identified by linear regression of experimental shear rheometry data using Matlab software (Mathworks, Natick, MA, USA); see “Results” section.

## Experimental Methods and Materials

In order to evaluate the novel CFD models against experimentally-observed flow, a series of fluids were created to span the range of IDDSI levels (from thinnest to thickest) and to span different types of fluids which could be encountered in dysphagia management: Newtonian liquids, and two types of liquids with different shear-thinning characteristics using starch-based and gum-based drinks thickeners. The CFD simulation was assessed against the experimental flow by comparing the changing volume of liquid in each case as well as the final volume of liquid at 10 s, which is the IDDSI flow test result.

### Thickened Drink Preparation

Powder quantities were measured (± 2 mg) using a precision electronic balance (AE Adam PGW 235e, Milton Keynes, UK). Liquids were pre-measured into 200 mL volumes (± 0.5 mL) in plastic beakers.

Starch-based Resource ThickenUp™ and xanthan gum-based Resource ThickenUp™ Clear (both Nestlé Health Science, Epalinges, Switzerland) were mixed to 200 mL Evian natural mineral water (Danone waters, Paris, France) at 21 °C at a range of concentrations from 0.125 to 4.5 g/100 mL. To ensure thorough dissolution samples were stirred vigorously for 20 s. To ensure the liquids were fully thickened they were left to thicken for 1 h before testing, then given a final brief stir and 1-min rest period.

Glycerol (>99%, Alfa Aesar, Heysham, UK) was diluted at a range of concentrations with distilled water and tested alongside drink thickeners to act as a Newtonian fluid comparison.

### IDDSI Flow Test and Video Image Analysis

All 10 mL syringes (Beckton-Dickson model 302188) were compliant with ISO standard 7886-1 and IDDSI specifications with a measured length of 61.5mm from zero line to 10 mL line. A 10 mL sample of each test liquid was filled slowly into a fresh syringe body, after removing its plunger. The nozzle was blocked using a gloved finger until the start of the flow test, at which point the nozzle was uncovered. The liquid drained into a beaker: these recovered samples were then tested on the rheometer, described later.

**Figure 2:**
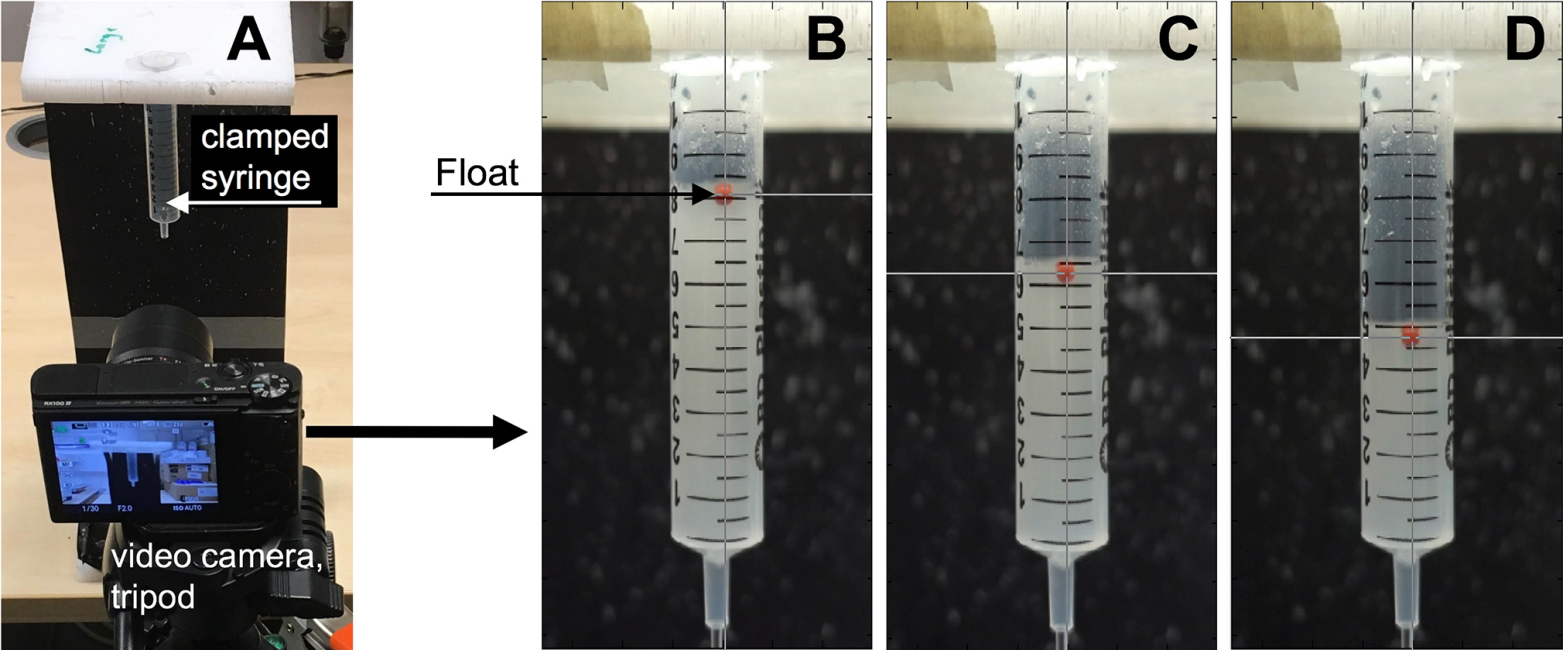
(a) Experimental setup for quantifying volume of liquid in the syringe by photogrammetry. (b, c, d) Representative images during analysis; the position of the red float marker is indicated at three example times (liquid volume = 8.1, 6.3 and 4.7 mL respectively).

The IDDSI flow test provides a single-point measurement of the fluid volume remaining in the syringe after a period of 10 s flow. In clinical practice a stopwatch is used for timing, but for increased accuracy in this study frame-by-frame video analysis was implemented. An experimental rig was constructed (Fig. 2) which provided standardized conditions for the IDDSI flow test, and provided continual measurement of the liquid volume throughout the 10 s duration. Liquid volume was measured by photogrammetry for minimal invasion of the fluid system. The presence of a meniscus means the surface level is indistinct, with uncertainty of approximately 0.3 mL on the syringe scale. To provide a higher-contrast surface marker, a 2.4 mm-diameter, bright red, expanded polystyrene bead was floated on the liquid surface using tweezers. A pilot test of 5 repeated measures of a Level 1 liquid using manual timings confirmed the float had no measurable or significant effect on the flow rate (*p* = 0.37). Digital video camera images (Sony RX100 M4 at 1920 × 1080 pixels, 50 frames per second) were analyzed using custom-written scripts in Matlab software (Mathworks, Natick, MA, USA) which located the position of this float marker to within 0.2 mm. Fluid volume measurements were recorded at 1 s intervals.

### Rheometry

Liquid samples (1.0 mL) were recovered after flow testing and measured on a rheometer (DHR-3, TA Instruments, New Castle, DE, USA) using cone and plate geometry (60 mm dia., 1° angle). A protocol was applied to assess yield and low-shear parameters before testing at higher shear rates:

1. A ramp of increasing shear stress from 0 until shear rate reached 1/s at which point this low-stress test was terminated.
2. Oscillatory testing was then performed at 1 Hz with a shear amplitude within the given fluid’s linear viscoelastic range (LVR), 0.01–0.5% depending on the material.
3. A shear ramp was performed with rate increasing from 0.1 to >1000/s.

## Results

### Rheology

**Figure 3:**
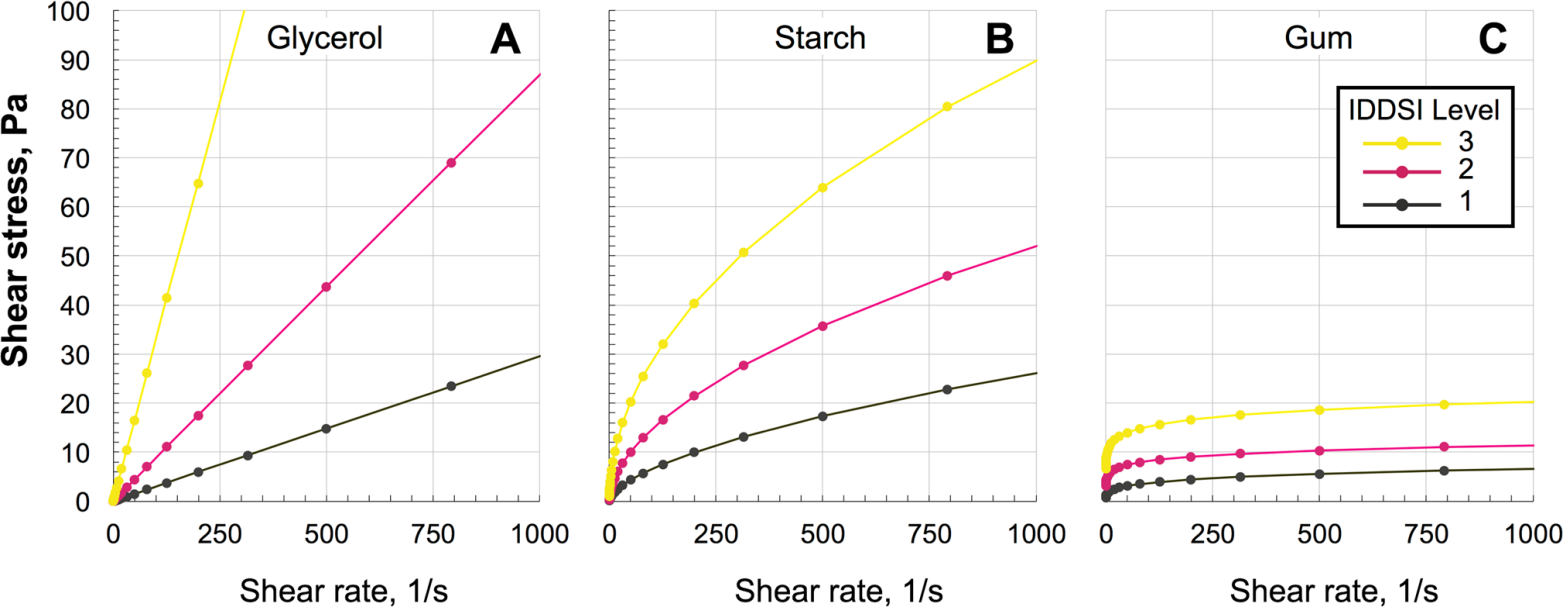
Measured shear rheology of the experimental materials: (a) Glycerol/water; (b) Starch-thickened water; (c) Gum-thickened water. Panels share a common *y*-axis; both axes are linearly-spaced.

Glycerol mixtures, being Newtonian, showed a linear relationship between applied stress and shear flow rate (Fig. 3a). However, starch- and gum-thickened water showed increasingly non-linear responses: for small increases in applied stress, these materials flowed disproportionately faster (Figs. 3b and 3c). For gum-thickened liquids, the relationship was so non-linear that the shear stress required to reach a fast shear rate of 1000/s in the thickest case, Level 3, was less than the stress required for the *thinnest* Level 1 liquids composed of starch or glycerol.

**Figure 4:**
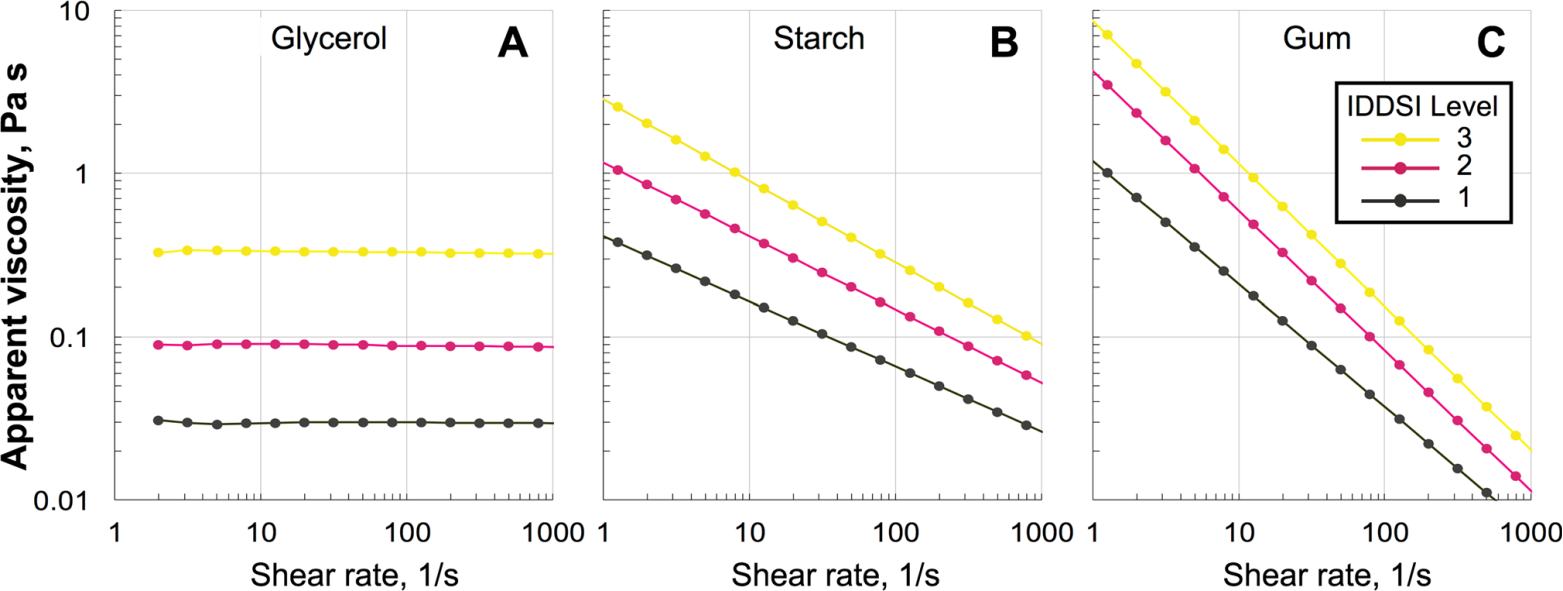
Shear rheology of the experimental materials: (a) Glycerol/water with constant viscosity; (b) Starch-thickened water, (c) Gum-thickened water, both with shear-thinning behavior. Panels share a common *y* axis; both axes logarithmically-spaced.

The apparent viscosity plots in Fig. 4 were derived from shear stress / shear rate. Newtonian glycerol mixtures had consistent viscosity irrespective of shear rate (Fig. 4a), but the apparent viscosity of starch- and gum-thickened water showed shear-thinning behavior (Figs. 4b and 4c), reflecting greater flow rate in response to incrementally increased stress.

Rheological models: A Herschel–Bulkley model was applied to the liquids^21^,^30^ using linear regression using Matlab software (Mathworks, Natick, MA).

5 $$ \tau = \tau_{y} + K\dot{\gamma }^{n} $$

6 $$ \ln \left( {\tau - \tau_{y} } \right) = \ln \left( K \right) + n { \ln }\left( {\dot{\gamma }} \right) $$

In which $\tau_{y}$ is the yield stress, $K$ is the consistency factor and $n$ is the power-law index. The best-fitting parameters for each liquid are tabulated in Table 1.

**Table 1:** Measured properties of fluid materials used in this investigation.

IDDSI level	IDDSI flow measure (mL)	Concentration to distilled water	Density (g/mL)	K (Pa s^n^)	n	Yield stress (Pa)	Apparent viscosity @50/s (Pa s)
Glycerol–water mixtures							
1	2.5	38 mL/1000 mL	1.01	0.0297	1.00	–	0.0297
2	6.0	200 mL/1000 mL	1.04	0.0885	1.00	–	0.0885
3	9.0	400 mL/1000 mL	1.07	0.327	1.00	–	0.327

IDDSI level	IDDSI flow measure (mL)	Concentration to Evian	Density (g/mL)	K (Pa s^n^)	n	Yield stress (Pa)	Apparent viscosity @50/s (Pa s)
Starch-thickened water							
1	2.5	3.37 g/100 mL	1.01	0.413	0.60	0.1	0.0867
2	6.0	4.05 g/100 mL	1.01	1.16	0.55	0.6	0.200
3	9.0	4.50 g/100 mL	1.02	2.86	0.50	1.0	0.404
Gum-thickened water							
1	2.5	0.70 g/100 mL	1.00	1.19	0.25	0.58	0.0627
2	6.0	1.13 g/100 mL	1.01	4.24	0.14	1.4	0.149
3	9.0	2.24 g/100 mL	1.01	8.65	0.12	3.4	0.280

### Measured vs. Simulated Volume of Liquid

**Figure 5:**
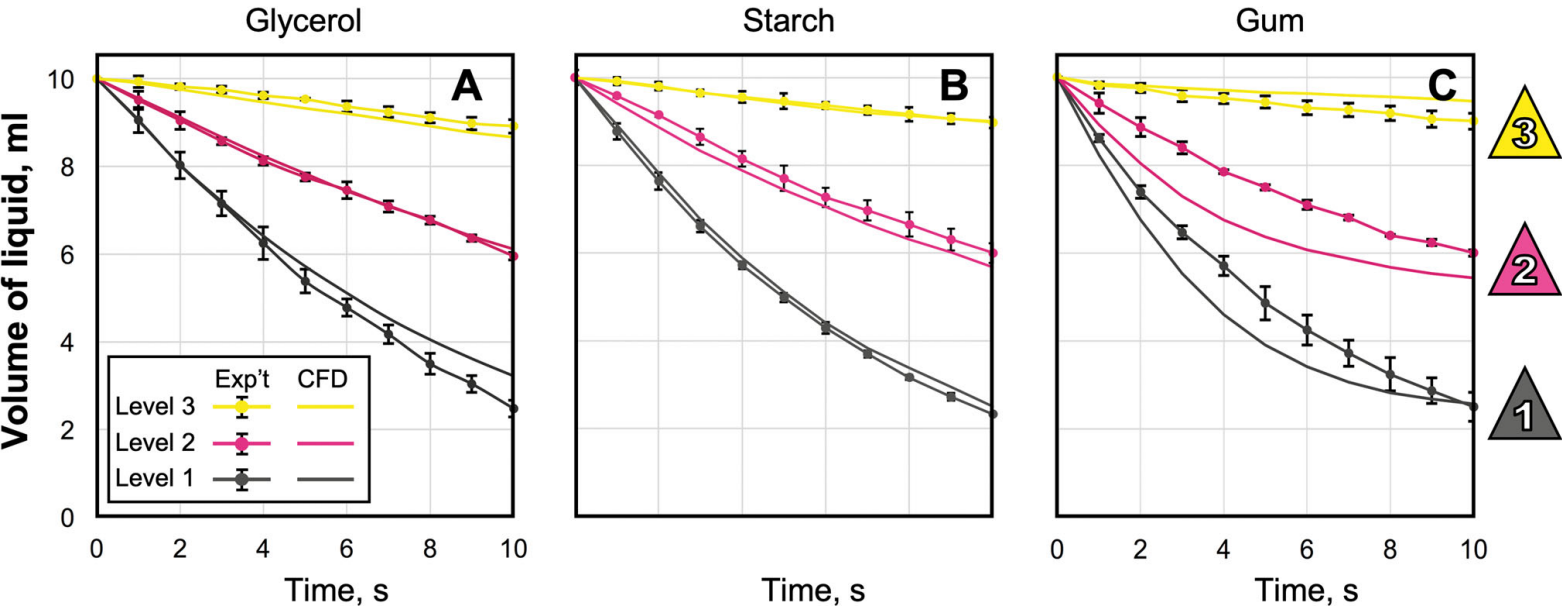
(a–c) CFD simulation of the volume of liquid remaining in the syringe barrel during the 10 s IDDSI flow test duration, in comparison with experimental data. Mean ± SD of the three repeated runs indicated by error bars. (a) Glycerol/water; (b) Starch-thickened water; (c) Gum-thickened water. Panels share a common *y* axis. IDDSI level labels are indicated on the right-hand side.

Figure 5 shows the volume of liquid in the syringe reducing as the liquid drains from the nozzle over the 10 s duration of the test (as measured by the image-tracking software). The CFD simulation of liquid volume is plotted on the same axes in each case. The simulations agreed well with the final values of the experimental IDDSI test. The deviation was less that 1 mL in absolute terms; in relative (%) terms, the deviation was largest for the thickest liquids (IDDSI Level 3), though the relative experimental variation (Coefficient of Variation, C.V.) was largest in these cases too. The trajectory of the gum-based liquids (Fig. 5c) showed a mis-match with the simulation over-estimating the rate of flow at the start of the tests, and under-estimating the rate of flow later. Although these effects act to partly cancel each other in the final result at 10 s, it is important to note the difference during the simulation.

**Figure 6:**
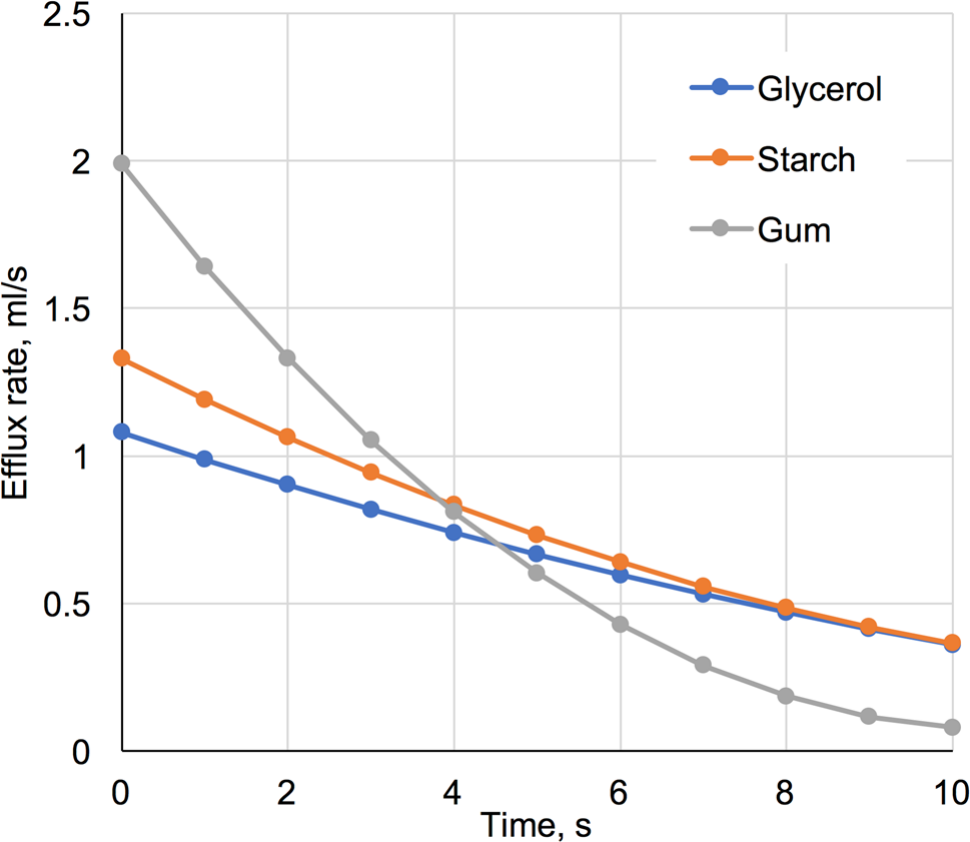
Simulated flow rate for three different types of IDDSI Level 1 liquids showing variation of efflux rate with time. All three have approximately the same total volume of efflux at the end of the 10 s period (all have approximately 2.5 mL remaining fluid at *t* = 10 s).

The rate of efflux varies over time, shown in Fig. 6. Initially the flow accelerates from stationary, however, since inertial forces are much lower than the viscous forces (see Mathematical Modelling section) the flow reaches maximum velocity very quickly: within the first 0.2 s. As the liquid height (hence, driving pressure) drops, the flow decelerates. As noted in Fig. 5c, the flow rates for gum appear to be over-estimated during the first half, and underestimated during the second half of the duration.

The results in Fig. 5 and Table 2 demonstrated that overall the computational models were able to simulate the final IDDSI test value observed experimentally, within the limitations identified above. The simulated flow was therefore deemed to be a sufficiently accurate representation of the IDDSI flow test to warrant further investigation into the *internal* conditions within the syringe. These are reported in the following section.

**Table 2:** CFD predictions and their comparison with experimental values for the final liquid volume of the three types of the liquids after 10 s of the IDDSI flow test.

IDDSI level	Glycerol mixtures			Starch mixtures			Gum mixtures		
	CFD	Exp. (C.V.)	Diff. (%)	CFD	Exp. (C.V.)	Diff. (%)	CFD	Exp. (C.V.)	Diff. (%)
1 [2.5 mL]	3.21	2.5 (3%)	+ 0.7 (9%)	2.33	2.5 (1%)	− 0.2 (2%)	2.56	2.5 (4%)	+ 0.1 (1%)
2 [6.0 mL]	6.11	6.0 (2%)	+ 0.1 (3%)	5.68	6.0 (6%)	− 0.3 (8%)	5.43	6.0 (2%)	− 0.6 (14%)
3 [9.0 mL]	8.68	9.0 (14%)	− 0.3 (32%)	8.98	9.0 (12%)	0.0 (0%)	9.46	9.0 (18%)	+ 0.5 (46%)

All values are in mL relating to the IDDSI flow test scale 0–10 mL. Percentages are calculated as a fraction of the volume of efflux (= 10 mL − final liquid volume) since the IDDSI scale operates in reverse, descending from 10 to 0

*C.V.* coefficient of variation of the experimental measurements, *Diff.* absolute difference between CFD and experimental values

### Velocity Profile and Shear Rate

**Figure 7:**
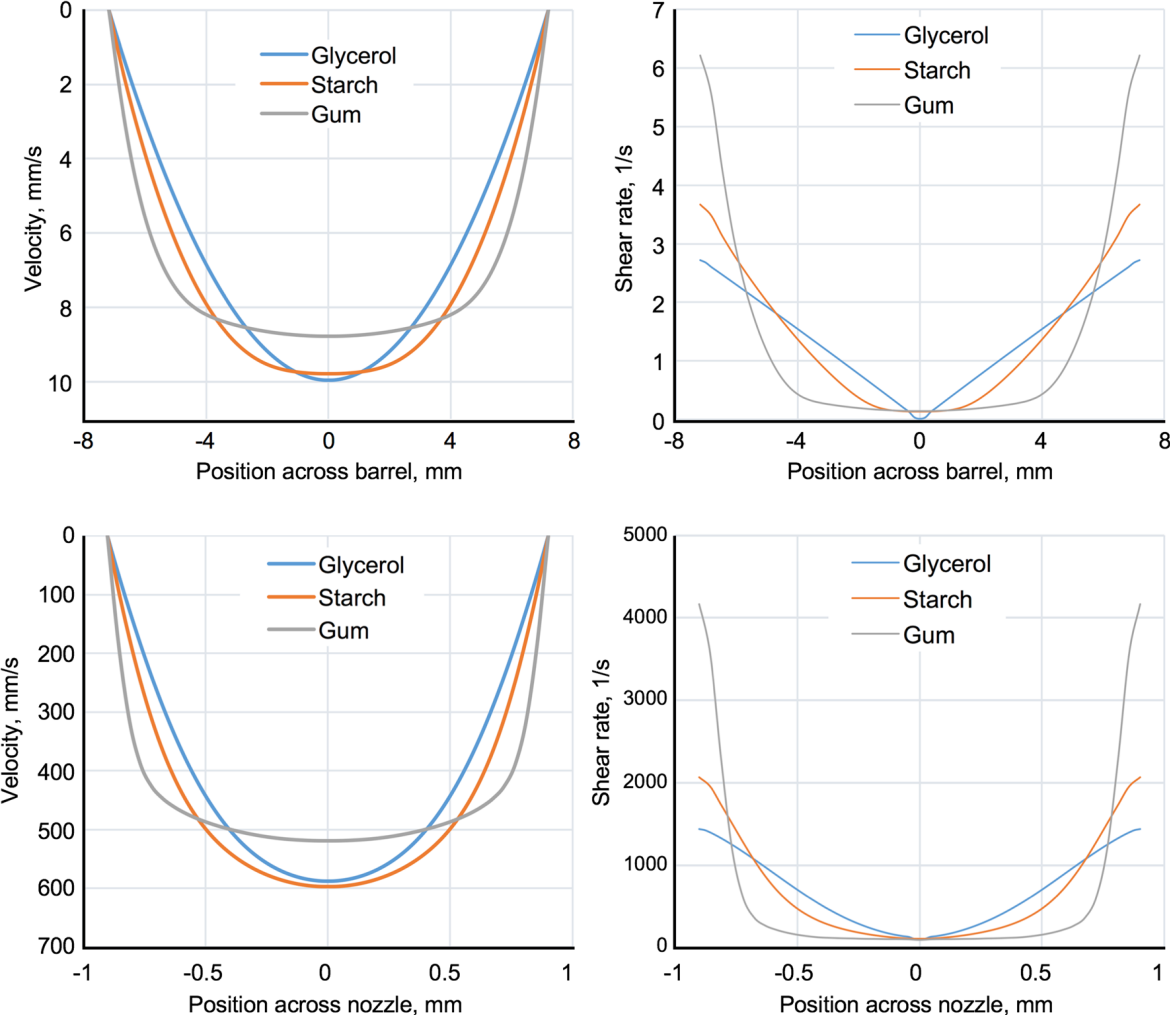
Computed flow velocity (Left) and shear rate (Right) in the syringe barrel (Top) and at the nozzle-end orifice (Bottom). IDDSI Level 1 liquids shown, at *t* = 3 s from start of test. Non-Newtonian (Gum and Starch) liquids show disproportionately higher shear rates at the edges, with higher maximum values than Newtonian (Glycerol).

Figure 7 (Left panels) shows the velocity profile of the fluids across the diameter of the barrel and nozzle of the syringe. The Newtonian Glycerol mixture matches the classical parabolic shape expected for flow in a pipe, and the non-Newtonian liquids show a “plug shaped” profile whereby the central portion flows at a relatively constant rate, which decreases down to zero across a relatively narrow region at the edges of the fluid. The Right Hand panels of Fig. 7 show the large differences in shear rate profiles for these different liquid types which are all at the same IDDSI consistency classification. Table 3 summarizes the values for *maximum* flow velocity and maximum shear rate in the barrel and nozzle. Differences between liquids are most pronounced at the thinnest IDDSI Level 1, where the rates of flow are highest. This corresponds to the deviation in the rheology of the liquids being greatest at these higher rates (Figs. 3a, 3b and 3c). In all cases, velocities at the nozzle orifice were much higher than the barrel, by a factor of approximately 60 since the cross-sectional area of the nozzle is approximately 1/60^th^ that of the barrel. As noted in Fig. 5c, the CFD simulated flow rate for the gum-based liquid appears to be an overestimation of the experimental observations, by up to 25%, which should be taken into account when interpreting these figures.

**Table 3:** Summary results from CFD simulation at maximum flow velocity, measured 1 s after the start of the test.

IDDSI level	Glycerol/ water	Starch	Gum
(A) Maximum velocity in the Barrel (mm/s)			
1	12.0	12.5	13.2
2	5.90	5.10	6.55
3	1.80	0.92	0.33
(B) Maximum velocity at the exit section of Nozzle (mm/s)			
1	695	737	746
2	372	350	432
3	116	63.8	17.8
(C) Maximum shear rate in the Barrel (1/s)			
1	3.30	4.60	9.32
2	1.60	2.45	7.12
3	0.50	0.51	0.30
(D) Maximum shear rate at the exit section of Nozzle (1/s)			
1	1790	2710	7400
2	833	1180	3880
3	258	233	185

## Discussion

CFD simulation based on rheological measurements was able to predict the experimentally-observed IDDSI flow test result of all fluid types and consistencies. On this basis, the simulations of *internal* flow patterns were assumed to be valid, however, it was not feasible to measure these experimentally for validation. These results showed parabolic flow of Newtonian liquids and “plug-shaped” flow of shear-thinning non-Newtonian fluids, as would be expected for flow through pipes. The extremely shear-thinning behavior of the Gum-thickened liquids (1.2 < *n* < 2.5) resulted in the shear rate being far higher at the walls than in the center: e.g. at the nozzle outlet (Fig. 6, lower Right panel) 90% of the shear occurred in just 22% of the radial distance. Consequently the maximum shear rate observed at the walls for Gum-thickened liquids was much higher than that for Starch or Glycerol mixtures having the same overall volumetric flow rate. This result confirms perceptual experiment results in which gum-based liquids are perceived as “slippery” in comparison to other types of thick liquid.^36^ This also concurs with the assertion that these types of liquid would be relatively suitable for successful swallowing.^36^

The hydrostatic pressure forcing the liquid out of the nozzle is proportional to the height of liquid in the syringe and the liquid density. In this study the density of liquids ranged from 1.00 g/cm^3^ (the thinnest Gum mixture) to 1.07 g/cm^3^ for the thickest glycerol mixture. The initial liquid height is equal for all liquids at the start of the test: 73 mm. Therefore, nominally, the maximum hydrostatic pressure would be 0.0731 m × 9.81 kg ms^−2^ × 1070 kg m^−3^ = 767 Pa. This is very low compared to tongue compression pressures measured *in vivo* during oral processing and swallowing which can be 1–2 orders of magnitude higher.^13^,^33^ However, that pressure was still sufficient to achieve high shear rates (max: 7400/s in the nozzle for the thinnest Gum-thickened liquid), at least as high as has been measured or simulated during swallowing.^25^,^27^,^31^ This implies that propelling these thickened liquids requires relatively very little tongue pressure in comparison to the pressure required to compress and swallow soft solid foods.^29^ Therefore these results suggest that liquids up to and including the IDDSI Level 3 classification would not require excessive effort to consume, despite their reduction in gravity-driven flow speed.

Regarding instances where the simulated flow results deviated from the experimental results, the deviation was greatest for the thickest (Level 3) liquids of all types (Table 2). However, at this consistency the experimental results also showed largest coefficient of variation, since the total efflux volume (1 mL) was smallest. None the less, there still appeared to be limitations in the simulation of the dripping-mode of outflow, whereby the surface tension effect had been approximated using a constant average back-pressure at the nozzle.^41^ In developing the computational model it was apparent that this back-pressure had a large effect on the simulated flow rate, suggesting that the drop formation mode has a significant influence on the outcome of the IDDSI flow test. Therefore, if a thick drink exhibited a very unusual magnitude of surface tension, it could affect the IDDSI flow test result. However, the same would apply for other rheometry techniques. The gum-thickened liquids in this study produced drops which stretched to a length of 5–10 times their diameter and then recoiled when a drop broke away. This cohesive characteristic was not included in the simulation: at the time of writing no commercially-available CFD solver includes elongational viscosity.

The relatively increased cohesiveness and elasticity of the gum-based liquids may also be responsible for the other instances where simulation differed from experimental results: the trajectories of the flow curves for all gum-based liquids in Fig. 5c. This may be attributable to limitations in the validity of the simulation’s no-slip assumption. The no-slip assumption may become invalid if the liquid has significant particle components: the particles near the boundaries may have limited interaction with the walls and a thin liquid layer on the wall may create an apparent partial slip condition,^39^ furthermore, partial slip is noted for polymeric solutions in micro-fluidic situations on a scale comparable to the size of the polymer.^34^ In the present studies, the no-slip assumption was applied on the basis that the liquids were observed to be homogeneous visually (i.e. there were no particles visible at scales comparable to the nozzle orifice) and the rheology measures had converged to a Herschel–Bulkley model (there was no longer evidence of partial slip with the rheometer surfaces). Further, the assumption was not expected to have a major effect on the results since the perimeter of the liquid-air interface was small compared to the rest of the contact surface between the liquid and the solid walls. However, the variable wall residue observed experimentally was a clear phenomenological discrepancy from the simulation’s uniform residual film, and was most pronounced for gum-based liquids. Therefore the combined effects of yield stress and partial wall slip may be worthy of investigation in future studies of these non-Newtonian liquids,^39^ and particularly if non-homogeneous suspensions are studied, e.g. partially-dissolved starch solutions or X-ray contrast media involving barium sulfate powder.

From the shear rheology properties given in Table 1 and Fig. 4 there is no simple shear measurement which predicts the result of the flow test. This is not surprising given the range of shear flow rates throughout the syringe (Fig. 6); previous studies of flow in simulation and *in vitro* have shown a broad distribution of shear rates occurring.^25^,^29^ Since the flow test is stress-controlled rather than strain-rate-controlled the shear rate varies depending on the fluid, being faster for thinner liquids. In this respect, the IDDSI flow test has some similarity with other practical measurement devices such as the Bostwick Consistometer and line-spread test (a form of slump test). Indeed, recent studies have directly compared the measurements achieved with these devices for a variety of fluids^8^,^15^ and found close-but-not-exact agreement since each test is dependent on slightly different fluid properties. In this work, the Herschel–Bulkley model (Eq. (5)) was not completely sufficient to describe the flow in the IDDSI flow test application. Therefore we can extrapolate that even this detailed liquid characterization would be incomplete as a description of texture-modified liquids for the management of dysphagia *in vivo*. Other researchers have hypothesized similarly and are investigating extensional viscosity, cohesiveness and other characteristics of fluids for dysphagia management.^11^,^35^,^40^

Instead of focusing on a specific rheological parameter (e.g. shear viscosity) which has been shown to be insufficient to describe flow in practice,^19^,^25^,^27^ the most fundamental property of any instrumental test is that the test condition is relevant to the application. In this case, *in vivo*, a bolus of liquid will experience a wide variety of shear rates during oral preparation and swallowing^25^,^31^; the shear rate will be maximal at the oropharyngeal surfaces and will always reach 0 in the center of the bolus. The IDDSI flow test represents a composite measurement of flow behavior at a wide range of velocities and shear rates: slower rates for thicker liquids and faster rates for thinner liquids. It is impractical to measure flow *in vivo*, but ultrasound Doppler measurements and simulations have provided evidence for slower flow with thicker liquids.^17^,^23^,^31^ That is the clinical intention in prescribing thickened liquids: that they would flow more slowly, providing the patient with more time to prepare for a swallow. It is therefore clinically relevant that the IDDSI flow test assesses rate of flow. A great deal of future research is required to identify clinical outcomes resulting from texture modification approaches; the wide availability of the IDDSI flow test may hopefully expedite that work.
